# Bond swapping from a charge cloud allows flexible coordination of upstream signals through WASP: Multiple regulatory roles for the WASP basic region

**DOI:** 10.1074/jbc.RA118.003290

**Published:** 2018-08-13

**Authors:** George J. N. Tetley, Aydan Szeto, Adam J. Fountain, Helen R. Mott, Darerca Owen

**Affiliations:** From the Department of Biochemistry, University of Cambridge, Cambridge CB2 1GA, United Kingdom

**Keywords:** CDC42, cell signaling, cytoskeleton, intrinsically disordered protein, protein conformation, protein motif, protein–protein interaction, small GTPase, structure–function, thermodynamics

## Abstract

Wiskott–Aldrich syndrome protein (WASP) activates the actin-related protein 2/3 homolog (Arp2/3) complex and regulates actin polymerization in a physiological setting. Cell division cycle 42 (Cdc42) is a key activator of WASP, which binds Cdc42 through a Cdc42/Rac-interactive binding (CRIB)-containing region that defines a subset of Cdc42 effectors. Here, using site-directed mutagenesis and binding affinity determination and kinetic assays, we report the results of an investigation into the energetic contributions of individual WASP residues to both the Cdc42–WASP binding interface and the kinetics of complex formation. Our results support the previously proposed dock-and-coalesce binding mechanism, initiated by electrostatic steering driven by WASP's basic region and followed by a coalescence phase likely driven by the conserved CRIB motif. The WASP basic region, however, appears also to play a role in the final complex, as its mutation affected both on- and off-rates, suggesting a more comprehensive physiological role for this region centered on the C-terminal triad of positive residues. These results highlight the expanding roles of the basic region in WASP and other CRIB-containing effector proteins in regulating complex cellular processes and coordinating multiple input signals. The data presented improve our understanding of the Cdc42–WASP interface and also add to the body of information available for Cdc42–effector complex formation, therapeutic targeting of which has promise for Ras-driven cancers. Our findings suggest that combining high-affinity peptide-binding sequences with short electrostatic steering sequences could increase the efficacy of peptidomimetic candidates designed to interfere with Cdc42 signaling in cancer.

## Introduction

The Ras superfamily consists of five families of monomeric G proteins with substantial structural homology and similarities in regulation. The ability to bind guanine nucleotides underlies the binary switch capacity of small G proteins and their role in transducing intracellular signals. Nucleotide exchange, leading to GTP binding in the nucleotide cleft, elicits conformational changes in the two switch regions of these proteins leading to activation, permitting interaction with effector proteins. Hydrolysis of GTP to GDP reverts the small G protein to an inactive state with corresponding changes in the switch regions. Nucleotide exchange and hydrolysis are promoted by guanine nucleotide exchange factors (GEFs)^3^ and GTPase-activating proteins (GAPs), respectively (1).

Cdc42 belongs to the Rho family of small G proteins of which many are key regulators of the actin cytoskeleton and therefore cell architecture and motility (2). Together with Rac1 and RhoA, Cdc42 constitutes the archetypal trinity of Rho G proteins that control the formation of actin-driven cellular structures such as lamellipodia, stress fibers, and filopodia. Often found to signal downstream of the master regulator small G protein Ras, Cdc42 induces the formation of filopodia by initiating actin remodeling, and this is key to cell motility. Deregulation therefore results in the progression of several disease states, including tumor metastasis (3). Genetic deletion of Cdc42 in Ras-transformed cells results in reductions in cell proliferation and cell cycle progression, demonstrating the essential role of Cdc42 in Ras-induced transformation (4). Accordingly, overexpression of Cdc42 is implicated in several human cancers and is correlated with poor disease outcome (5–7). Although mutations in Cdc42 are rarely observed in human cancers, oncogenic mutations have been characterized in its regulators such as the GEFs Dbl (8) and Asef2 (9) and the GAP DLC1 (10). Activating and inactivating mutations in GEFs and GAPs, respectively, increase the basal level of Cdc42·GTP contributing to cellular transformation. Cdc42 participates in both physiological and tumorigenic processes by interacting with effector proteins. One such family of effectors, the CRIB proteins, is defined by a consensus sequence contained within their Cdc42-binding region and includes activated Cdc42-associated kinase (ACK), the p21-activated kinases (PAKs), and Wiskott–Aldrich syndrome proteins (WASPs) (11).

We have previously solved structures of Cdc42 bound to the G protein–binding domains (GBDs) of ACK and PAK1 (12, 13). Structures are also available for Cdc42 bound to WASP (14), PAK6 (PDB 2ODB), and PAK4 (15). The GBDs of these effectors reside within intrinsically disordered regions of the proteins, which become structured, to varying degrees, on binding to Cdc42. We, and others, have also undertaken thermodynamic studies of these complexes, including our recent complete survey of the Cdc42–ACK interface (16). Additionally a growing body of data is available regarding the mechanism of binding adopted by these CRIB effectors. It is becoming increasingly clear that the family utilizes a “dock-and-coalesce mechanism” of binding, with WASP and PAK1 possessing electrostatic steering regions to mediate docking (17–19), whereas ACK exploits hydrophobic contacts to direct complex formation (16). The biophysical analyses of these complexes generate data that can be used to drive rational drug design, by informing peptide therapeutics engineering and by providing data pinpointing the relevant part of chemical space to target with small molecules.

Blockading Cdc42 signaling should have therapeutic efficacy. Nur-E-Kamal *et al.* (20) demonstrated that introduction of the ACK GBD into Ha-Ras–transformed NIH3T3 cells reversed the classic metrics of transformation, presumably by blocking signaling downstream of Cdc42. Recent advances in peptide chemistry have made the use of peptide inhibitors more feasible, which coupled to their exquisite specificity, good biocompatibility, and ease of synthesis, makes the use of peptide inhibitors an attractive therapeutic option (21, 22). Our recent mutational analysis of the ACK GBD identified several key residues that mediate Cdc42 binding, including the consensus CRIB residues (16). These data indicated which regions of the ACK GBD contain the energetically important determinants for high affinity Cdc42 binding and should therefore be contained within a therapeutic peptidomimetic.

**Figure 1.**
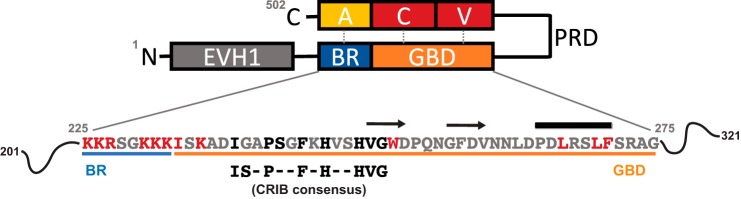
**Domain architecture of WASP.** Structural domains and regions with assigned function of WASP are highlighted as follows: the Ena/VASP homology domain 1 (*EVH1, gray*); the basic region (*BR, blue*); the G protein-binding domain (*GBD, orange*); the central proline-rich domain (*PRD*); the verprolin homology (*V, red*); central (*C, red*); and acidic region (*A, yellow*). WASP exists in an autoinhibited conformation when not bound by effectors, and the BR and A regions and the CV and GBD regions form contacts that prevent the VCA region binding and activating Arp2/3. Only WASP residues 225–275, which include the BR and residues of the GBD mutated in this study, are expanded to show their sequence *below*, and the residues selected for mutagenesis are *highlighted*. Residues colored *black* are CRIB consensus residues, which were all subject to mutation in this study; residues outside the CRIB region mutated in this work are colored *red.* The CRIB consensus sequence is also included. Secondary structure elements in WASP when bound to Cdc42 are shown *above* the sequence. β-Strands are denoted by *arrows* and the helix by a *cylinder*. The limits of the secondary structure elements are taken from Ref. 14 with amendments included from Ref. 29. The accession number for WASP is UniProt no. P42768.

Similar to ACK, the WASP proteins, WASP and N-WASP, are also CRIB family Cdc42 effectors. WASPs are key regulator of the Arp2/3 complex and therefore drivers of actin assembly (23). WASP-mediated actin polymerization is coordinated by the C-terminal verprolin homology, central, and acidic (VCA) region, which interacts with both the Arp2/3 complex and monomeric actin (Fig. 1). The WASP–GBD is located between the N-terminal Ena/VASP homology domain 1 (EVH1) and the central proline-rich domain (PRD). In the autoinhibited state, the VCA region is shielded by the GBD via intramolecular interactions, until Cdc42·GTP activates WASP by disrupting these autoregulatory interactions through complexing with the WASP–GBD (23). There are interesting differences, however, between the ACK GBD- and WASP GBD–Cdc42 interactions. First, a basic region (BR) precedes the WASP CRIB region; this is absent in the ACK counterpart. In addition to stabilizing the aforementioned autoinhibitory interactions, these basic residues have been proposed to play an electrostatic steering role in Cdc42 recognition, acting as a specificity determinant for Cdc42 over Rac1 (17). Second, and perhaps consequently, Cdc42 has a significantly higher binding affinity for WASP (*K_d_* 1 nm) compared with ACK (*K_d_* 30 nm) (24).

The high affinity for Cdc42 achieved by the WASP GBD implies that it would be a useful starting point for designing a peptide therapeutic targeting Cdc42. Despite several extremely useful biophysical studies on WASP in the past, to our knowledge a systematic thermodynamic and kinetic analysis of the WASP GBD binding to Cdc42 has never been undertaken. These intriguing qualities of WASP motivated us to undertake a more comprehensive analysis of the Cdc42–WASP complex, to reaffirm and augment our previous analyses using the ACK GBD, as well as dissecting the role of individual residues in the WASP BR in regard to specific and rapid Cdc42 recognition, leading to the identification of further properties for inclusion in therapeutic peptides.

## Results

### Design of mutations in the WASP GBD

Several sources of information guided the design of WASP mutations. We have previously performed a mutational analysis of Cdc42 with regard to binding of CRIB effectors, including WASP, and so we incorporated residues on WASP likely to interact with energetically important residues on Cdc42 (24). We also analyzed the structure of the Cdc42–WASP GBD complex and identified residues in WASP that were within 3.5 Å of Cdc42 residues (Ref. 14 and PDB code 1CEE). Leu-267, Leu-270, and Phe-271 in the C-terminal helix of the GBD were identified as candidates due to their proximity to a hydrophobic pocket of Cdc42. Conserved residues of the WASP CRIB consensus motif (25) were obvious candidates for mutation: Ile-238, Pro-241, Phe-244, His-246, His-249, Val-250, and Gly-251. As mutations in the equivalent ACK residues severely impaired Cdc42 binding, it was expected that the CRIB residues would play similar roles in WASP and therefore have similar effects on Cdc42 binding upon mutation (16). Residues outside the CRIB region, analogous to those identified by our recent study on the ACK GBD (16), were also selected (Ile-233, Lys-235, and Tyr-252). Finally, a series of positively charged residues (Lys-225, Lys-226, Arg-227, Lys-230, Lys-231, and Lys-232) that constitute the WASP basic region (BR) were included to dissect their potential roles in both the final Cdc42–WASP complex and in the steering of the encounter complex of the two proteins. 20 residues in total were identified for mutational analysis, as shown in Fig. 1 and Table 1. All residues were mutated to alanine, a small, neutral residue, which allows the calculation of the energetic contribution of individual side chains to the binding interface. Mutations were introduced into a construct expressing WASP residues 201–321 (26). This construct is long enough to include all parts of WASP likely to interact with Cdc42. Most work published using WASP fragments that include this region report very little structure in the free WASP peptides (14, 26), which only include a small amount of α-helix. Alanine is not predicted to affect helicity as it has the highest helical propensity of the amino acids (27). Therefore, although we cannot rule out that the mutations could affect residual secondary structure present in uncomplexed WASP, it is unlikely.

**Table 1:** **WASP residues mutated in this study**

WASP residue	Location	Interaction partner(s) on Cdc42
Lys-225	Basic region	*^a^*
Lys-226	Basic region	*^a^*
Arg-227	Basic region	*^a^*
Lys-230	Basic region	*^a^*
Lys-231	Basic region	*^a^*
Lys-232	Basic region	*^a^*
Ile-233	Cdc42/WASP structure	Ile-46, Leu-174, Leu-177
Lys-235	Basic region	Lys-153, Glu-171, Leu-174
Ile-238	CRIB residue	Val-44, Ile-46, Asp-170, Ile-173, Leu-174
Pro-241	CRIB residue	Tyr-23, Val-42, Val-44, Lys-166
Ser-242	Cdc42/WASP structure	Val-42, Thr-43
Phe-244	CRIB residue	Thr-24, Thr-25, Tyr-40, Val-42
His-246	CRIB residue	Tyr-40
His-249	CRIB residue	Asp-38
Val-250	CRIB residue	Val-36, Phe-37
Gly-251	CRIB residue	*^b^*
Trp-252	Cdc42/WASP structure	Val-36
Leu-267	Cdc42/WASP structure	Phe-37, Leu-70
Leu-270	Cdc42/WASP structure	Leu-70
Phe-271	Cdc42/WASP structure	Phe-37, Leu-67, Leu-70

*^a^* Residues of the basic region do not appear to bind to specific residues on Cdc42 but rather make nonspecific interactions with negatively charged residues in the vicinity.

*^b^* Gly-251 is a CRIB residue that does not appear to interact specifically with any Cdc42 residues in the Cdc42/WASP structure.

### Binding affinity analysis

**Figure 2.**
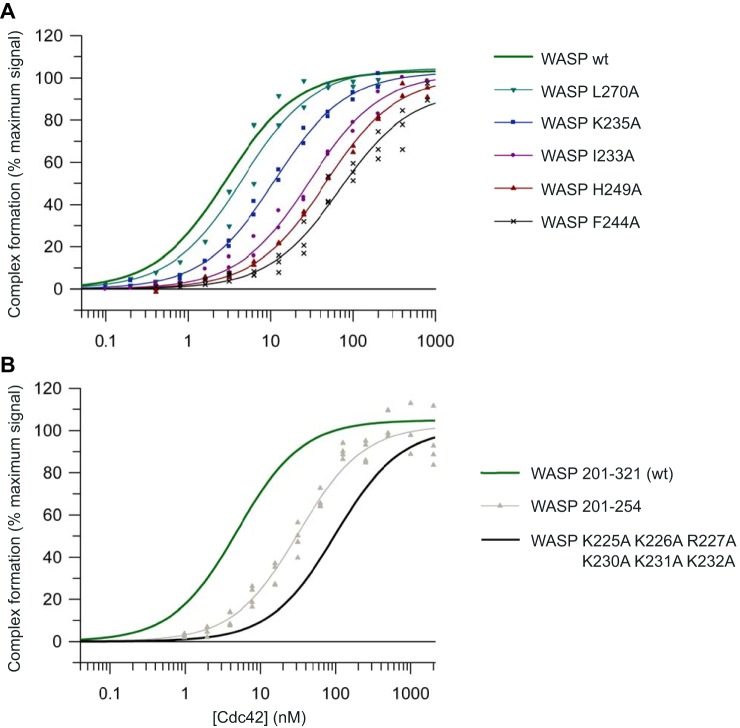
**Direct SPA-binding data for the WASP GBD and mutant variants, with Cdc42.** The indicated concentration of [^3^H]GTP-labeled Cdc42 was incubated with GST-tagged WASP GBD variants, as appropriate. The SPA signal was corrected by subtraction of the background signal from parallel measurements in which the effector protein was omitted. The effect of the concentration of Cdc42 on this corrected SPA signal was fitted to a binding isotherm to give an apparent *K_d_* value and the signal at saturating Cdc42 concentrations. The data and curve fits are displayed as a percentage of this maximal signal. *A,* binding of representative mutants in the WASP GBD to Cdc42. *B,* binding of WASP BR hexa-mutant and C-terminal deletion mutant to Cdc42. 2–4 experimental replicates were performed for each WASP variant with 12 data points in each. A summary of all the binding data can be found in Table 2.

First, the energetic contributions of the 14 residues that reside in the GBD were assessed. The apparent *K_d_* values for the interaction between the WASP mutants and Cdc42 were determined by direct scintillation proximity assays (SPAs). Example binding isotherms are shown in Fig. 2*A*, and the affinities are summarized in Table 2. Consistent with our previous analysis (24), the WT WASP–GBD interacts with Cdc42·GTP with high affinity, having a *K_d_* of 0.5 nm. All 14 alanine substitutions led to decreased affinity for Cdc42.

**Table 2:** **Equilibrium binding constants and thermodynamic data for WASP GBD mutants**

Mutation	*K_d_**^a^*	-Fold increase	Δ*G*	ΔΔ*G*
	*nm*		*kcal/mol*	*cal/mol*
WT	0.5 ± 0.1		−12.4	
I233A	26.4 ± 2.4	52.8	−10.1	2.3
K235A	8.0 ± 0.6	16.0	−10.8	1.6
I238A	ND*^b^*	>2000*^c^*		
P241A	38.3 ± 3.0	76.6	−9.9	2.5
S242A	6.4 ± 0.9	12.8	−10.9	1.5
F244A	64.9 ± 7.7	129.8	−9.6	2.8
H246A	42.3 ± 4.5	84.6	−9.8	2.6
H249A	43.1 ± 2.3	86.2	−9.8	2.6
V250A	10.1 ± 1.1	20.2	−10.7	1.7
G251A	5.2 ± 0.5	10.4	−11.0	1.4
W252A	9.6 ± 0.9	19.2	−10.7	1.7
L267A	3.2 ± 0.5	6.4	−11.3	1.1
L270A	1.7 ± 0.4	3.4	−11.7	0.7
F271A	5.0 ± 0.8	10.0	−11.1	1.3
201–254	31.8 ± 2.7	63.6	−10.0	2.4

*^a^* The standard error from curve fitting is shown.

*^b^* ND (no binding) denotes data that could not be fitted to the binding isotherm.

*^c^* We assume a *K_d_* of >1 μm (the limit of accurate direct SPA calculations).

### Contribution of CRIB consensus residues to the Cdc42–WASP complex

**Figure 3.**
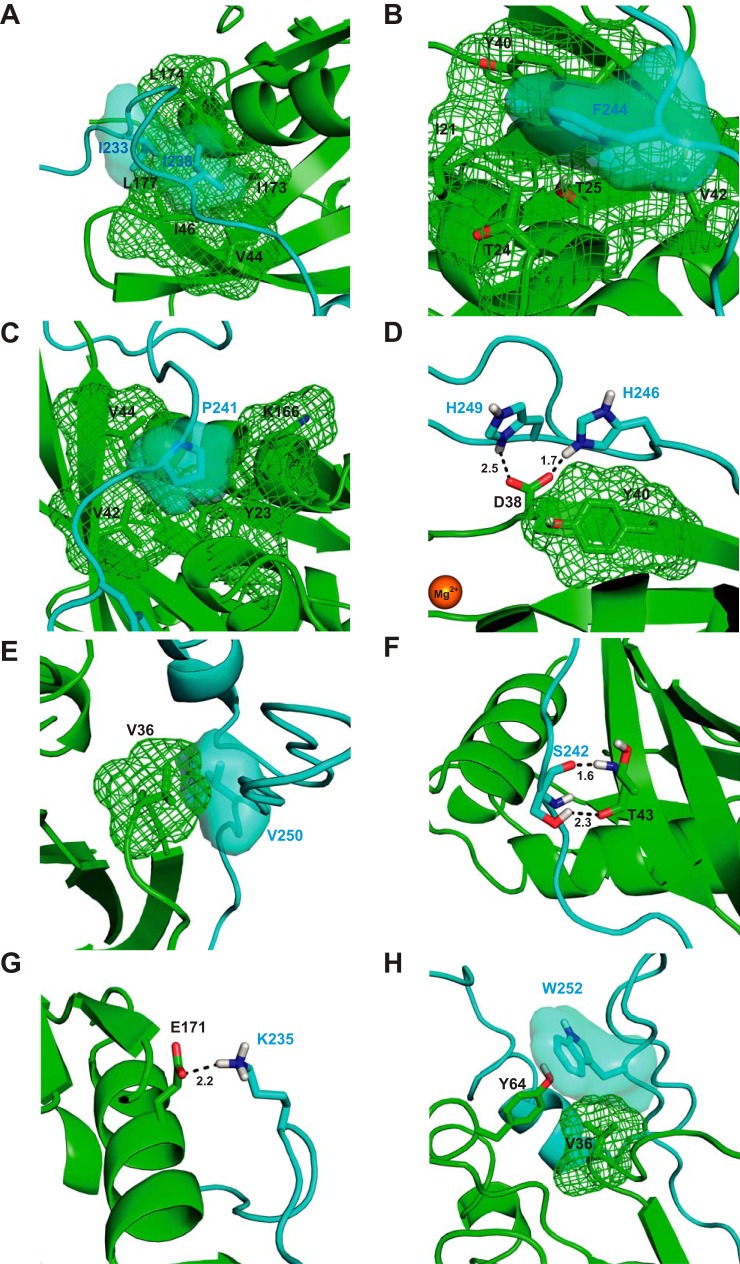
**Structural analysis of mutational effects.** *A,* schematic representation of part of the Cdc42/WASP–GBD structure (Ref. 14 and PDB 1CEE) showing the contacts made by Ile-233 and Ile-238^WASP^. The van der Waals surfaces of relevant residues are shown either as a *mesh* or a *semi-transparent surface* (using the PyMOL default solvent radius of 1.4 Å). Cdc42 is colored *green*, and WASP is colored *blue. B*, schematic representation of part of the Cdc42/WASP–GBD structure showing the contacts made by Phe-244^WASP^. Coloring is as in *A. C,* schematic representation of part of the Cdc42/WASP–GBD structure showing the contacts made by Pro-241^WASP^. Coloring is as in *A. D*, schematic representation of part of the Cdc42/WASP–GBD structure showing the contacts made by His-246^WASP^ and His-249^WASP^. Coloring is as in *A. E,* schematic representation of part of the Cdc42/WASP–GBD structure showing the contacts made by Val-250^WASP^. Coloring is as in *A. F,* schematic representation of part of the Cdc42/WASP–GBD structure showing the contacts made by Ser-242^WASP^. Coloring is as in *A. G,* schematic representation of part of the Cdc42/WASP–GBD structure showing the contacts made by Lys-235^WASP^. Coloring is as in *A. H,* schematic representation of part of the Cdc42/WASP–GBD structure showing the contacts made by Trp-252^WASP^. Coloring is as in *A*. Where highlighted, oxygen atoms are colored *red* and nitrogen in *blue*. Magnesium is shown as an *orange sphere*.

Out of the 14 mutants investigated, five exhibited dramatic reductions in affinity for Cdc42 of more than 70-fold. All five correspond to mutations at CRIB consensus positions, which is consistent with both their conservation and our similar previous mutational analysis of the CRIB effector protein, ACK (16). The parallels between the WASP and ACK GBDs are perhaps most evident from the essential roles of WASP Ile-238 and the equivalent ACK Ile-454 in Cdc42 binding. Ile-238 is the first consensus residue in the WASP CRIB region (Fig. 1), and mutation decreases Cdc42 binding to the extent that a *K_d_* cannot be accurately determined in this assay, suggesting that this residue contributes >35% of the binding energy to the interface (Table 2). A similar decrease in affinity is seen when mutating Ile-454 to alanine in ACK (16). Ile-238^WASP^ interacts substantially with a number of hydrophobic residues of Cdc42 (Ile-173, Ile-46, and Val-44), as seen in the Cdc42–WASP structure (Fig. 3*A*). Mutation of isoleucine into the much smaller alanine would extensively reduce the strength of hydrophobic interaction within the pocket. The central importance of Ile-238^WASP^ may also be justified as this first N-terminal CRIB residue may be essential in initiating the coalescence phase of GBD binding after initial docking. This has been postulated for ACK (16), as the residue is involved in extensive hydrophobic contacts with a less flexible region of Cdc42 giving a platform for continued quaternary structure formation after an initial docking phase.

The F244A mutation has the second largest impact in terms of decreasing Cdc42 affinity, causing an ∼130-fold increase in *K_d_* (Table 2). Phe-244^WASP^ is the fourth consensus residue of the WASP CRIB region (Fig. 1). The effects of F244A can be explained by the extensive hydrophobic contacts that Phe-244 makes with Cdc42 residues. The residue inserts between α1 and β3 in Cdc42 where it is involved in a π stacking interaction with Tyr-40^Cdc42^ and also contacts the γ carbon of Thr-25^Cdc42^ (Fig. 3*B*). The effect of the F244A mutation is also consistent with previous mutational analysis; removal of the tyrosine ring in the Y40C^Cdc42^ mutation causes a dramatic 830-fold decrease in affinity for WASP (24), and the F462A mutation in ACK causes a similarly large decrease in affinity (16).

P241A, H246A, and H249A mutations also led to major increases in *K_d_* (∼77-, 85- and 86-fold, respectively) (Table 2). Pro-241^WASP^ supports a number of hydrophobic interactions with Cdc42 residues, occupying a hydrophobic cleft between α1, β1, and α6, proximal to residues such as Val-42/44^Cdc42^ and also interacting with the side chains of Tyr-23^Cdc42^ and Lys-166^Cdc42^ (Fig. 3*C*). This residue forms, and potentially stabilizes (due to the conformational restrictions of proline), a characteristic kink in the binding conformation of WASP and indeed other CRIB effectors. In the Cdc42–ACK structure, this allows the ACK peptide to penetrate the Cdc42 structure more deeply, imbuing the analogous proline in ACK with an even greater effect on binding affinity.

His-246^WASP^ and His-249^WASP^ are both proximal to Asp-38^Cdc42^, and the latter is close to Tyr-40^Cdc42^ (Fig. 3*D*). The Asp-38^Cdc42^ carboxylate group has the potential to form hydrogen bonds to the side chain –NH of His-246^WASP^ and His-249^WASP^; these may well also be exchangeable given the variable orientation of these histidines in the family of structures (PDB code 1CEE). The calculated ΔΔ*G* for both H246A and H249A is ∼2.6 kcal mol^−1^ (10.9 kJ mol^−1^), which is within the range of the strength of a single hydrogen bond (5–30 kJ mol^−1^). His-249^WASP^ is often oriented in parallel with the aromatic ring of Tyr-40^Cdc42^ in the family of structures, which would indicate a possible π-stacking interaction; however, given the similarity of the effect of mutation of either histidine residue, a model where His-246^WASP^ and His-249^WASP^ exchange interactions, principally with Asp-38^Cdc42^, is appealing.

Alanine mutations of the remaining two consensus CRIB residues, Val-250 and Gly-251, have more modest effects on Cdc42 binding of ∼20- and 10-fold increase in *K_d_* compared with WT. Val-250^WASP^ packs close to Val-36^Cdc42^ in the complex structure, likely supporting hydrophobic contacts (Fig. 3*E*). Gly-251^WASP^ does not appear to interact specifically with any Cdc42 residues; none are within 3.5 Å. This gives rise to the possibility that this small residue may function in providing flexibility to the WASP GBD (and indeed the ACK GBD (16)) rather than participating in specific interactions. Substitution with a larger residue could result in some steric interference in the binding complex, thus raising the *K_d_*.

Ser-242 mutation resulted in a 12.8-fold drop in affinity. Interestingly, this has a smaller impact on binding than might be expected given its effect on ACK binding (over 200-fold). The side chain of Ser-242^WASP^ is correctly oriented to form a hydrogen bond to Thr-43^Cdc42^, but it is possible that this interaction could be replaced by the backbone amide hydrogen in the Ala-242 variant, perhaps explaining the less dramatic reduction in affinity (Fig. 3*F*).

### Energetic contribution of residues N-terminal to the CRIB consensus region

Mutation of residues N-terminal to the CRIB consensus has moderate effects on Cdc42 binding, including Ile-233 (53-fold decrease) and Lys-235, (16-fold decrease). Ile-233 has been identified as a specificity determinant for ACK, defining it as a Cdc42-only effector (16, 24), and it is probable that the residue plays a similar role in WASP. Ile-233 forms contacts with Leu-174/177^Cdc42^ (Fig. 3*A*), and this patch of hydrophobicity is not shared by small GTPases closely related to Cdc42 such as Rac1 or TC10.

Its position toward the N terminus of the WASP GBD suggests Lys-235 may contribute to the positive charge in this region and thus to the electrostatic attraction to Cdc42 supported by the BR (see below); however, because the residue is slightly separate from the densely charged region between residues 225 and 232, its influence is likely to be relatively small. Furthermore, the 16-fold decrease in affinity is considerably larger than that caused by alanine substitution of individual BR residues (see below), implying that Lys-235 has a more specific role in the Cdc42–WASP structure. The residue is oriented close to Glu-171^Cdc42^, with which it could form a hydrogen bond (Fig. 3*G*).

### Residues C-terminal to the CRIB consensus sequence

Mutation of residue Trp-252 to alanine results in a 19.2-fold decrease in affinity for Cdc42. Trp-252 is positioned in the Cdc42–WASP structure so as to form hydrophobic interactions with Val-36^Cdc42^ and a hydrogen bond with the side chain –OH of Tyr-64^Cdc42^ (Fig. 3*H*).

Residues Leu-267^WASP^, Leu-270^WASP^, and Phe-271^WASP^, located C-terminal to the CRIB consensus region, appear to cover a hydrophobic pocket formed by Val-36^Cdc42^, Phe-37^Cdc42^, Leu-67^Cdc42^, and Leu-70^Cdc42^ in the Cdc42–WASP structure. Single mutations of L267A, L270A, and F271A have relatively small effects, suggesting that individually these residues contribute little to Cdc42 binding. The hydrophobic nature of alanine is however unlikely to be particularly disruptive to such interactions. Hydrophobic interactions C-terminal to the CRIB consensus residues in the Cdc42–ACK complex have a similar contribution to binding energy (16). It is possible that residues in this region of WASP collectively provide a hydrophobic patch that acts as a final anchor point for the GBD; however, this is clearly not as influential to binding as elements of the CRIB region.

### Contribution of the WASP basic region to the Cdc42–WASP complex

**Figure 4.**
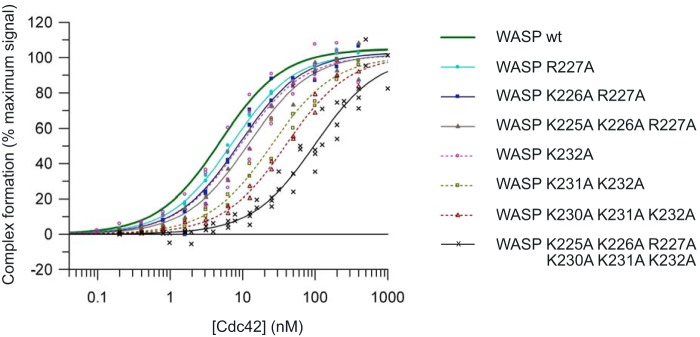
**Direct SPA-binding data for the WASP GBD and mutant BR variants, with Cdc42.** The indicated concentration of [^3^H]GTP-labeled Cdc42 was incubated with GST-tagged WASP GBD BR variants, as appropriate, in each SPA. The SPA signal was corrected by subtraction of the background signal from parallel measurements in which the effector protein was omitted. The effect of the concentration of Cdc42 on this corrected SPA signal was fitted to a binding isotherm to give an apparent *K_d_* value and the signal at saturating Cdc42 concentrations. The data and curve fits are displayed as a percentage of this maximal signal. 2–4 experimental replicates were performed for each WASP variant with 12 data points in each. A summary of all the binding data can be found in Table 3.

The contribution of the basic region of WASP to the electrostatic steering of the encounter complex with Cdc42 is well documented (17, 18). However, we still lack a systematic analysis of all individual residues and all combinations of residues in the BR to both the overall affinity of the Cdc42–WASP complex and to the on-rate of complex formation. To address this, we made a further set of mutations and combinations of mutations in the WASP BR. The BR comprises two triads of positive residues, Lys-225–Arg-227 and Lys-230–232; these were mutated singly, in pairwise combinations within each triad, and then each complete triad individually and together. We first analyzed their binding to Cdc42 in direct SPAs: example binding isotherms are shown in Fig. 4, and the affinities are summarized in Table 3.

**Table 3:** **Equilibrium binding constants and thermodynamic data for WASP BR mutants**

Mutation	*K_d_**^a^*	-Fold increase	Δ*G*	ΔΔ*G*
	μ*m*		*kcal/mol*	*cal/mol*
WT	0.5 ± 0.1		−12.4	
K225A	2.8 ± 0.3	5.6	−11.4	1.0
K226A	5.4 ± 0.8	10.8	−11.0	1.4
R227A	4.3 ± 0.4	8.6	−11.1	1.2
K225A/K226A	7.4 ± 1.2	14.8	−10.8	1.6
K225A/R227A	7.5 ± 1.0	15	−10.8	1.6
K226A/R227A	5.7 ± 0.7	11.4	−11.0	1.4
K225A/K226A/R227A	8.8 ± 1.0	17.6	−10.7	1.7
K230A	9.7 ± 0.7	19.4	−10.7	1.7
K231A	12.4 ± 1.0	24.8	−10.5	1.9
K232A	12.2 ± 1.0	24.4	−10.5	1.8
K230A/K231A	12.9 ± 1.2	25.8	−10.5	1.9
K230A/K232A	18.4 ± 2.1	36.8	−10.3	2.1
K231A/K232A	21.7 ± 2.8	43.4	−10.2	2.2
K230A/K231A/K232A	36.0 ± 3.1	72	−9.9	2.5
K225A/K226A/R227A/K230A/K231A/K232A	98.6 ± 10.9	197	−9.3	3.1

*^a^* The standard error from curve fitting is shown.

The SPA data (Table 3) show that K225A, K226A, R227A, K230A, K231A, and K232A single mutations had relatively small effects on Cdc42 binding (6–25-fold increase in *K_d_* compared with WT). Structural data are only available for the second triad in the BR, residues 230–232, as the WASP fragment used for the published structural studies comprised residues 230–288 (14). Similar affinity measurements for residues 230–232, both singly and in combinations, can be reconciled both with the lack of specific interactions in the structure for Lys-230^WASP^, Lys-231^WASP^, and Lys-232^WASP^ in the Cdc42–WASP complex and with the model that these residues are primarily involved in encounter complex recognition through forming a positive charge cloud, complementary to negative residues on Cdc42. From the single mutation data, residues in the C-terminal triad of the BR appear to contribute slightly more to the overall affinity of the complex, with mutation of these residues resulting in a slightly lower affinity than single mutations in the N-terminal triad. Data on all possible combinations of single residues of the BR show a similar pattern: double and triple combination mutations of the N-terminal triad result in a smaller decrease in *K_d_* than substitutions made in the C-terminal triad, with replacement of all three N-terminal positive residues (225–227) with alanine resulting in an increase in affinity of 18-fold as compared with 72-fold for the C-terminal triple substitution. Replacement of all six positive residues of the BR with alanine resulted in a reduction in affinity of 197-fold. This loss of affinity is higher than that seen with any mutation in the consensus CRIB residues, except for Ile-238 (Table 2). The loss of the C-terminal BR triad alone has an effect comparable with most CRIB consensus residues (and lowers the *K_d_* to a value close to that of the ACK GBD for Cdc42), demonstrating the importance of the BR to high-affinity WASP binding.

These data suggest that the residues toward the C terminus of the BR (and therefore closer to the final complex interface) contribute more to the overall *K_d_* of WASP for Cdc42; although contributions are modest on an individual level, removal of all positive residues has a significant effect on stability of the final complex. This suggests the requirement for a cloud of positive potential on WASP in this region.

### Association (k_on_) and dissociation (k_off_) rate constants for Cdc42–WASP

**Figure 5.**
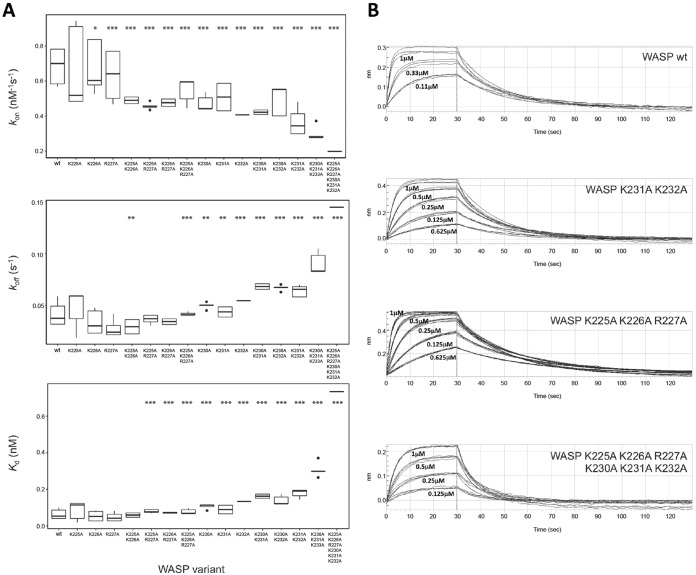
**Binding kinetics for WASP BR mutants measured by bio-layer interferometry.** GST fusion proteins were loaded onto anti-GST sensors and dipped alternately into a concentration range of Cdc42·GMPPNP (of at least three different concentrations) and buffer to measure on- and off-rates. *A,* data for *k*_on_, *k*_off_, and *K_d_*, where *n* = 3–9 independent experiments, plotted as *boxplots* showing median and quartile values with data outlying the distribution appearing as dots for all mutants. Statistical significance from WT values is indicated as follows: *, *p* ≤ 0.05; **, *p* ≤ 0.01; ***, *p* ≤ 0.001 as analyzed by *t* test with the false discovery rate controlled by the Benjamini-Hochberg method (analysis performed in R). Note: K225A/K226A value is statistically significantly lower than the WT but not than the other single mutations in the N-terminal triad; therefore, this is most likely a false discovery error. *B,* raw data from representative experiments. The fitted values from these data are reported in Table 4.

To address the potential roles of the BR residues in the electrostatic steering of the encounter complex between WASP and Cdc42, we analyzed the kinetics of the interaction between our panel of basic region variants and Cdc42 using biolayer interferometry (BLI). BLI has the advantage over SPA of yielding the dynamic rate constants *k*_on_ and *k*_off,_ from which the *K_d_* is calculated. If residues in the BR primarily function in electrostatic steering, then a decreased *k*_on_ for the variants would be expected compared with WT, whereas *k*_off_ should be unaffected (however, overall *k_a_* might be affected due to changes in rebinding). The Lys/Arg-Ala mutants should therefore display similar *k*_off_, with reduced *k*_on_ principally accounting for the corresponding increases in *K_d_* observed in SPAs. A summary of the kinetic data for all BR mutants is shown in Table 4 and Fig. 5*A*, with representative association and dissociation curves shown in Fig. 5*B*.

**Table 4:** **Binding kinetics for WASP variants with Cdc42**

Mutation	*k*_on_*^a^*	*k*_off_	*K_d_*
	μ*m*^−*1*^ *s*^−*1*^	s^−*1*^	n*m*
WT	0.691 ± 0.096	0.0417 ± 0.0113	63.7 ± 26.2
K225A	0.671 ± 0.215	0.0468 ± 0.0168	83.5 ± 45.2
K226A	0.676 ± 0.136	0.0338 ± 0.0106	54.4 ± 24.7
R227A	0.630 ± 0.144	0.0281 ± 0.0086	49.0 ± 23.2
K225A/K226A	0.490 ± 0.019	0.0295 ± 0.0073	59.6 ± 12.4
K225A/R227A	0.456 ± 0.017	0.0371 ± 0.0040	81.5 ± 8.7
K226A/R227A	0.497 ± 0.018	0.0372 ± 0.0004	74.8 ± 2.8
K225A/K226A/R227A	0.540 ± 0.065	0.0420 ± 0.0014	79.2 ± 12.2
K230A	0.477 ± 0.042	0.0498 ± 0.0029	105.5 ± 12.2
K231A	0.509 ± 0.082	0.0438 ± 0.0052	89.6 ± 24.5
K232A	0.407 ± 0.020	0.0546 ± 0.0008	134.0 ± 6.8
K230A/K231A	0.422 ± 0.014	0.0684 ± 0.0028	162.5 ± 12.0
K230A/K232A	0.484 ± 0.077	0.0670 ± 0.0026	141.8 ± 23.3
K231A/K232A	0.368 ± 0.004	0.0639 ± 0.0001	177.7 ± 0.5
K230A/K231A/K232A	0.300 ± 0.039	0.0914 ± 0.0096	307.4 ± 37.3
K225A/K226A/R227A/K230A/K231A/K232A	0.198 ± 0.019	0.1450 ± 0.0034	734.0 ± 72.6

*^a^* The mean values for *k*_on_, *k*_off_, and *K_d_* with standard errors from three independent experiments are shown.

The *K_d_* value obtained for WT WASP with Cdc42 by BLI was 63.7 nm (Table 4), which is approximately 2 orders of magnitude greater than that obtained by SPA. In general, the *K_d_* values obtained for all six single mutants were the same as, or slightly higher than, that of WT (Table 4). The affinities for variants with combinations of the N-terminal BR triad residues removed are also close to WT. Mutation of combinations of the C-terminal trio of basic residues resulted in larger decreases in affinity, with mutations of all six residues giving a *K_d_* of ∼730 nm. These data reflect the same trends as seen in the SPA data, although the fold changes are lower.

The differences in absolute values obtained by SPA and BLI likely stems from two factors. First, these are quite different analytical systems and data fitting methods, which would not be expected to produce the same actual dissociation rate values. Second, and potentially more importantly for this system, the buffer used in the BLI and SPA experiments differed. SPA data were collected in a low-salt buffer. BLI experiments were conducted in 150 mm NaCl to minimize nonspecific binding effects. Although the NaCl concentration used is close to physiological levels, the differences caused by charge–charge interactions will be lower, as noted in previous experiments on the WASP BR (17). With these differences in mind, the relative changes between mutants are used to drive conclusions here, rather than absolute values.

The *k*_on_ and *k*_off_ values for the WASP BR mutants show interesting trends with charge depletion in each of the BR triads. The association rate for WT WASP and Cdc42 is 0.691 μm^−1^ s^−1^ and the dissociation rate is 0.042 s^−1^. As alanine is substituted for the basic residues, the on-rate drops; this change is small and, although statistically significant for all mutations apart from K225A, likely physiologically insignificant for the individual mutations in the N-terminal triad. The decrease in on-rates becomes more significant (statistically and physiologically) with the increased number of mutations. The effects are greater for the C-terminal triad residues, which indeed show as much of an effect when singly mutated as the N-terminal triad triple mutant. The completely substituted hexa-mutant gives the lowest on-rate, ∼3.5-fold lower than WT.

Interestingly, and unexpectedly, the off-rates of the C-terminal triad mutants are also affected, with incrementally modest yet significant increases with increasing charge depletion, resulting in an overall doubling of the off-rate in the triple mutant. Such *k*_off_ differences are not observable in mutants of the N-terminal BR; however, mutation of all six basic residues to alanine increases the off-rate by 3.5-fold, more than the C-terminal triad alone, suggesting the N terminus does affect off-rate in certain situations. These data indicate that the BR of WASP is not only involved in electrostatic steering of complex formation but that it also has an influence on final complex stability.

### Overall contribution of the C terminus of the WASP GBD to Cdc42 binding

The Cdc42-binding region of WASP diverges from ACK both at the BR and the region C-terminal to the CRIB. Since the GBD of ACK appears to have higher affinity than WASP when the BR is neutralized, the increased affinity must come either from the slight differences in the configuration of ACK around the CRIB region or from the C terminus. To investigate this, a WASP mutant was expressed lacking GBD residues C-terminal to Pro-254 (WASP(201–254)), equivalent to a truncated mutant of ACK used in our previous investigations (16). Binding was tested to Cdc42 under exactly the same conditions as those used to study ACK (16). Results are summarized in Table 2, and the data are shown in Fig. 2*B*. These show that the C terminus of WASP contributes less than the BR to the overall binding affinity for Cdc42, with a 64-fold decrease in affinity. Compared with ACK, where deletion of the C-terminal GBD causes a 238-fold reduction in binding affinity to Cdc42, the equivalent region of WASP, despite being longer, is 4-fold less influential (16).

## Discussion

**Figure 6.**
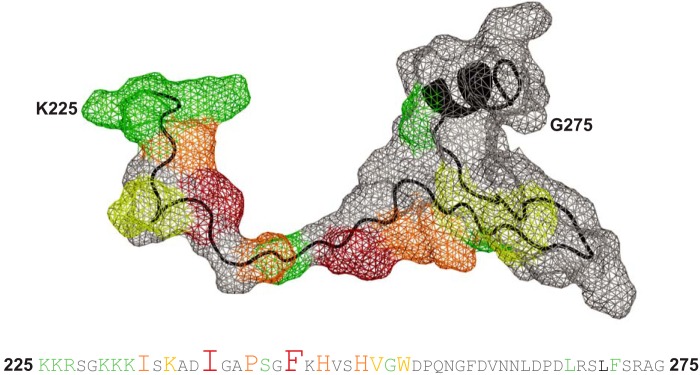
**Energetic contribution of individual WASP residues to Cdc42 binding.** The *upper panel* shows the van der Waals surface of WASP residues 225–275 as a *gray mesh* with the protein backbone in *black.* Residues involved in interaction with Cdc42 are colored with respect to the fold-change in *K_d_* upon mutation to alanine as follows: *green* for 5–15-fold increase; *yellow* for 15–40-fold increase; *orange* for 40–100-fold increase; and *red* for a >100-fold increase. The amino acid sequence for this region is shown in the *lower panel*, with residues colored in the same manner and with font size also indicating relative energetic contribution to complex formation. The accession number for WASP is P42768.

The mutational analysis of WASP residues in relation to Cdc42 binding presented here confirms the likely dock-and-coalesce mechanism of interaction between the two partners, with docking driven by electrostatic steering. Energetic hotspots on WASP for interaction with Cdc42 were identified in the study, and in a similar scenario to that found in ACK, the highly contributing residues are centered around the N terminus of the CRIB region. Fig. 6 shows the relative importance of individual residues for binding, mapped onto the structure of WASP and in the sequence.

Our data are largely in agreement with previous analysis of the electrostatic steering mechanism of Cdc42–WASP (17), although the scale of effects we observe are more modest than those previously reported. This likely reflects small differences in reagents and the different methodologies utilized in the respective analyses. Both sets of data (SPA and BLI) suggest a role for electrostatic steering in Cdc42–WASP recognition, with residues in the C-terminal triad of the BR having larger contributions. However, in all cases the largest effects are observed when multiple mutations are introduced, suggesting that a cloud of positive potential in this region is required for steering.

Unexpectedly, our data reveal a concomitant increase in the off-rate of the Cdc42–WASP complex when mutations are introduced into the C-terminal triad of BR residues, indicating that these residues likely play a significant role in the final binding complex of Cdc42–WASP. The effect of alanine mutations within these three residues depends on the overall charge loss rather than the precise position, implying that these residues underpin a nonspecific ionic interaction. Hemsath *et al.* (17) suggest that these residues interact with the oppositely charged Glu-49 and Glu-178 on Cdc42. The construct used in their work and in the Cdc42–WASP structure (14) is missing Glu-181^Cdc42^, which could also add to the ionic interactions between Cdc42 and the Lys-230–232^WASP^ triad. In contrast, mutation of the N-terminal triad of BR residues has little effect on the off-rate of the complex, implying that these residues do not contribute to ionic interactions. It is, however, noteworthy that the off-rate of the K225A/K226A/R227A/K230A/K231A/K232A mutant is higher than that of the K230A/K231A/K232A mutant. This suggests that the positive residues in the N-terminal triad of the BR can compensate, to some extent, for loss of the charges when the C-terminal triad is mutated. This region of WASP is likely to be unstructured, potentially facilitating this compensation, which is lost when all of the positive residues are removed. Since the N-terminal triad residues have no effect on the off-rate in the presence of an intact C-terminal triad, it should be noted that this effect is unlikely to come into play in a natural setting with PIP_2_ and other potential charge interactions present. It does, however, illustrate that the BR increases the binding ability of CRIB effectors, both for increasing the efficacy of the dock-and-coalesce mechanism and for increasing the stability of the final construct. Mutations in the C-terminal triad, however, combinations of which cause a greater than 2-fold decrease in *k*_on_ and a concomitant 2-fold increase in *k*_off_, are likely to have physiological significance in Cdc42 binding. The complete neutralization of BR residues, which causes the largest changes in *k*_on_ and *k*_off_ would certainly be expected to significantly affect the activation of Arp2/3 in cells, as WASP binding to Cdc42 would be shorter in duration due to the combined decreased in on-rate and increase in off-rate. Thus, it appears that the BR assists in facilitating a robust actin polymerization response. The high association rate of Cdc42–WASP has been shown to be acutely important for WASP's ability to stimulate actin polymerization (17).

The results from this analysis suggest that Lys-235 of WASP functions in a different manner to lysine residues between positions 225 and 232. K235A has a significantly larger effect on Cdc42–WASP equilibrium binding than point mutations of BR residues, is among the N-terminal residues of the GBD (between Ile-233 and Ile-238), and is well resolved in the structure of Cdc42–WASP. An initial observation into the effect of a K235A mutation on the binding kinetics of Cdc42–WASP suggests that mutation affects the off-rate of WASP more than individual mutations of 230–232 (data not shown). This reinforces the hypothesis that Lys-235 forms stable interactions in the final complex rather than simply contributing to a steering charge cloud. As such, the residue should be considered part of the GBD, not the BR, although this does not preclude a minor contribution from Lys-235 to the positive charge cloud in this region of WASP.

The complete analysis of the thermodynamic contribution of residues of the WASP GBD to complex formation with Cdc42 suggests an overall association mechanism similar to that found in Cdc42–ACK (16). After steering, we envisage the hydrophobic contributions from the residues immediately C-terminal to the BR providing the anchor point for the interface, with Ile-238 of the CRIB consensus being the key residue. Ile-238 importantly binds to residues on Cdc42 outside the flexible switch regions. This would drive further interactions through the CRIB region involving Pro-241, Ser-242, Phe-244, His-246, His-249, Val-250, and Gly-251, constituting the coalesce phase of the interaction. As in ACK, residues in WASP would position and stabilize the switch loops of Cdc42 in the active conformation. The final stages of the coalesce phase would involve hydrophobic interactions with residues C-terminal to the CRIB consensus, *e.g.* Trp-252 and Phe-271, which would act to stabilize and anchor the C-terminal end of the interface in a similar manner to the situation in Cdc42–ACK (16).

**Figure 7.**
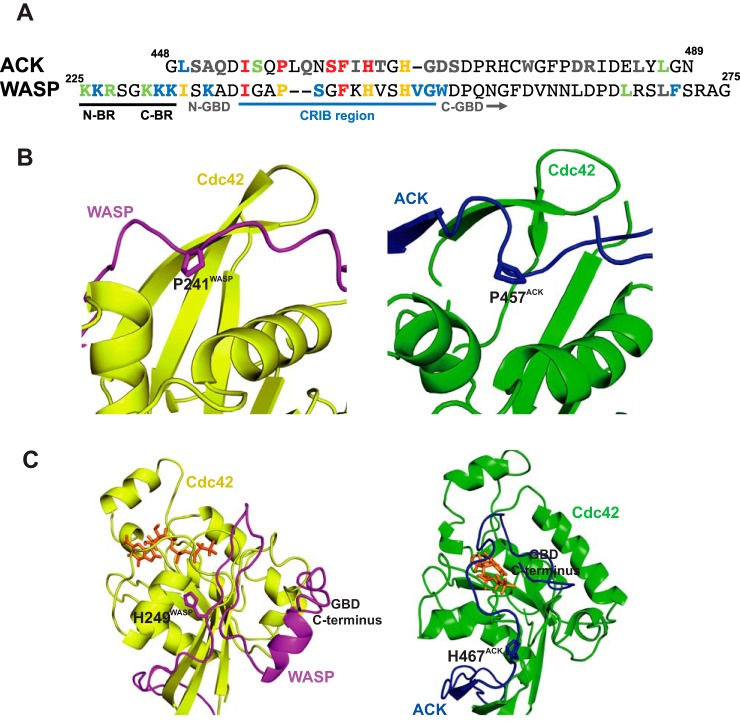
**Key differences in ACK and WASP binding to Cdc42.** *A,* alignment of the ACK GBD and WASP BR–GBD sequences. The UniProt accession number for WASP is P42768 and for ACK is Q07912. Residues are colored according to their effect on equilibrium constant: *gray,* 0–5-fold increase; *green,* 5–10-fold increase; *blue*, 10–50-fold increase; *orange*, 50–100-fold increase; *red*, 100 or greater fold increase. The CRIB region, N- and C-terminal BR triads, and N- and C-terminal regions of the GBDs are labeled. *B,* schematic representation of part of the Cdc42–WASP GBD (*yellow/magenta*, PDB code 1CEE (14)) and Cdc42–ACK GBD (*green/cyan*, respectively, PDB code 1CF4 (12)) structures highlighting Pro-241^WASP^ and Pro-457^ACK^. *C,* schematic representation of part of the Cdc42–WASP GBD (*yellow/magenta*, PDB code 1CEE (14)) and Cdc42–ACK GBD (*green/cyan*, respectively, PDB code 1CF4 (12)) structures highlighting the positions of the C-terminal regions of the WASP and ACK GBDs. Coloring is as in *B*. The nucleotide is shown as a *stick* representation, colored *orange*.

It is noticeable that the affinity of the WASP GBD with a charge-depleted BR is around 4-fold lower than that of ACK. The GBDs of these effectors vary principally in two places, ACK has a two-residue insertion in the middle of the CRIB consensus (Fig. 7*A*), and there is little homology between the two proteins after the last His residue of the CRIB. Although the positioning of the CRIB residues is generally conserved between ACK and WASP in complex with Cdc42, there appears to be a difference in the depth of insertion into the Cdc42 structure centered around Pro-457^ACK^ and Pro-241^WASP^. The extra two residues in ACK allow the CRIB motif to insert deeper into the hydrophobic pocket in Cdc42 than can WASP (Fig. 7*B*). The fold change for the proline mutation in ACK is around double that for WASP (191- and 76-fold, respectively). Mutation of Ser-461^ACK^ has a 20-fold greater effect than Ser-242^WASP^; therefore it is possible that the greater GBD affinity of ACK for Cdc42 could stem in part from differences in this region.

The C-terminal section of the WASP GBD, however, has a lower affinity for Cdc42 than the analogous region in ACK, and the chain positions of WASP and ACK with respect to Cdc42 also differ here (Fig. 7*C*). Given that the difference in affinity is around 4-fold, this appears to account for the 4-fold poorer binding of WASP to Cdc42 than ACK in the absence of the BR. This part of the WASP sequence is also involved in WASP autoinhibition and binds to the VCA region; the evolution of multiple roles for this region may have resulted in a decreased ability to bind Cdc42.

### WASP activation in cells

Research over the past 2 decades has revealed an exquisite orchestration of the regulation of actin remodeling directed by WASP proteins. The WASP proteins themselves serve as platforms to integrate multiple signal inputs, with their activity controlled by numerous ligands and through multiple mechanisms with resulting WASP activation stimulating actin polymerization by Arp2/3 (28).

Unstimulated WASP exists in an autoinhibited conformation where the VCA region is bound by part of the GBD region (Ref. 29 and PDB code 1EJ5). NMR studies show that the structured region in the inhibited form of WASP spans WASP residues His-249 to Glu-295, which include the end of the CRIB consensus and the C-terminal region of the WASP GBD (Fig. 7). The BR residues are not included in this globular, structured region (PDB code 1EJ5) nor are the N-terminal WASP GBD residues that, as we report here, are critical for Cdc42 binding, such as Ile-233, Ile-238, Pro-241, and Phe-244. However, the BR residues are thought to interact with the acidic region of WASP/N-WASP (30, 31) due to complementary charge interactions, and studies using truncated constructs suggest there are more extensive interactions than those identified in the available structure (32).

The VCA domain needs to be released from the autoinhibited complex to interact with Arp2/3 and stimulate actin polymerization. Among other potential signals (see later discussion), the binding of Cdc42 to the GBD causes destabilization of the WASP autoinhibited structure, releasing the VCA and thus stimulating polymerization (32). The BR seems a likely candidate for initiating this transition. Interaction with an alternative negative charge density, such as that on Cdc42, would allow exchange of the BR–acidic region interactions, weakening the overall structure of autoinhibited WASP and opening up access to the N-terminal GBD residues for further interaction with Cdc42. The GBD residues could then exchange cis-interactions for those with Cdc42 and release the VCA domain. Since our data suggest that initial docking relies on nonselective charge interactions, conformational sampling by the GBD, which should be limited in the autoinhibited structure, would be unnecessary. It would therefore be expected that release of the inhibitory GBD from the VCA would be significantly less likely without the presence of a BR, and this sequence is likely to act as the initiating switch between binding partners for the GBD.

**Figure 8.**
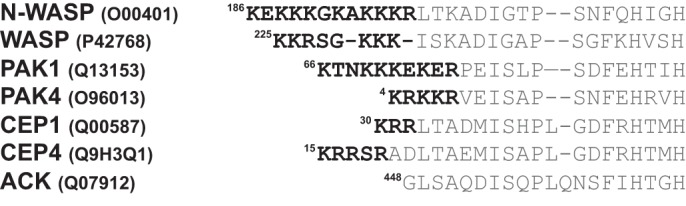
**Alignment of the basic regions of CRIB effector proteins.** Accession numbers for all proteins are shown, as are starting residue numbers. Basic region residues are highlighted in *black*.

PIP_2_ is also known to play a role in the activation of the WASP family. Most of the research into the role of PIP_2_ has used N-WASP, which possesses a slightly larger BR than WASP (Fig. 8). N-WASP has been shown to be synergistically activated by PIP_2_ and Cdc42 *in vitro* (33). Moreover, a further study (30) showed that N-WASP is multivalent for PIP_2_ binding, which is cooperative in WASP activation, in part because the BR of N-WASP appears again to be a constituent of the auto-inhibition complex. Cdc42 significantly changes this cooperativity between N-WASP and PIP_2_, allowing lower levels of PIP_2_ to activate N-WASP. Such observations imply that Cdc42 and PIP_2_ bind to N-WASP simultaneously to relieve VCA autoinhibition, the obvious mechanism being where Cdc42 binds the GBD and some or all of the BR interacts with PIP_2_. Notably the affinity of Cdc42 for full-length WASP is significantly lower than that of the BR–GBD expressed alone, due to the formation of the autoinhibited structure with the VCA region (32). The system used here, without the VCA region, allows us to probe Cdc42 binding effectively (and gain more accurate binding data for this region) and assess individual residue contributions to this individual structure. The BR may, however, be even more important in the transition between the autoinhibited structure and the Cdc42-bound form, whereas certain GBD residues may make important contacts in the autoinhibited WASP structure thus having an effect on affinity for Cdc42. This makes it likely that in a physiological setting WASP activation is reliant on external factors such as membrane localization (by WIP and TOCA) (34) and other activating stimuli such as PIP_2_ (30).

Naturally, this model, where PIP_2_ binds the BR, would aid in membrane localization of N-WASP. PIP_2_ tends to be localized to certain areas of the membrane in nanoclusters (35), and considering that higher local densities of PIP_2_ activate N-WASP (30), such sites could provide loci for actin nucleation, even in the absence of Cdc42. Membrane localization also increases the chance of WASP encountering active Cdc42, which is similarly membrane-localized, in the cell.

WASP has a similar sequence to N-WASP (Fig. 8), and one would expect delocalized charge interactions in the BR to be similar between the related proteins. PIP_2_ has been shown to activate WASP, similarly to N-WASP, *in vitro* (29), and other research has suggested that Arp2/3 activation is enhanced by WASP interaction in a system with both PIP_2_ and active, prenylated Cdc42 (36). Interestingly, in this study Cdc42 alone did not stimulate actin polymerization, despite the significant binding interface between Cdc42 and regions of the WASP that have been reported to be part of the autoinhibited conformation (Ref. 29 and PDB code 1EJ5).

The simultaneous binding of PIP_2_ and active membrane-localized Cdc42 by WASP appears likely given the data in this study, particularly because the N-terminal region of the BR does not appear to be involved in Cdc42–WASP binding, leaving it available for PIP_2_ interaction. Since the interactions of the BR are electrostatic, they are likely to be quite fluid and, as such, the residues of the BR could switch interactions between the acidic region of WASP, PIP_2_, and Cdc42, with the C-terminal BR triad not forming stable contacts in the WASP–Cdc42 complex.

Since the on- and off-rates of WASP binding to Cdc42 are high, individual interactions are likely to be transient. However, the simultaneous interaction of WASP with PIP_2_ and active Cdc42 could stabilize WASP in an open form by reducing the off-rate from the complex. Simultaneous binding at several linked sites is known to produce such an effect (37). The proximity of the BR and the GBD should strengthen the off-rate effect and reduce Cdc42–WASP unbinding events. Stable release of the VCA should increase the duration and robustness of Arp2/3 activation, producing more and longer actin filaments. Future experiments could explore the dependence of PIP_2_, or indeed another anionic lipid, together with Cdc42 in WASP activation and the effects of simultaneous binding on the off-rate.

**Figure 9.**
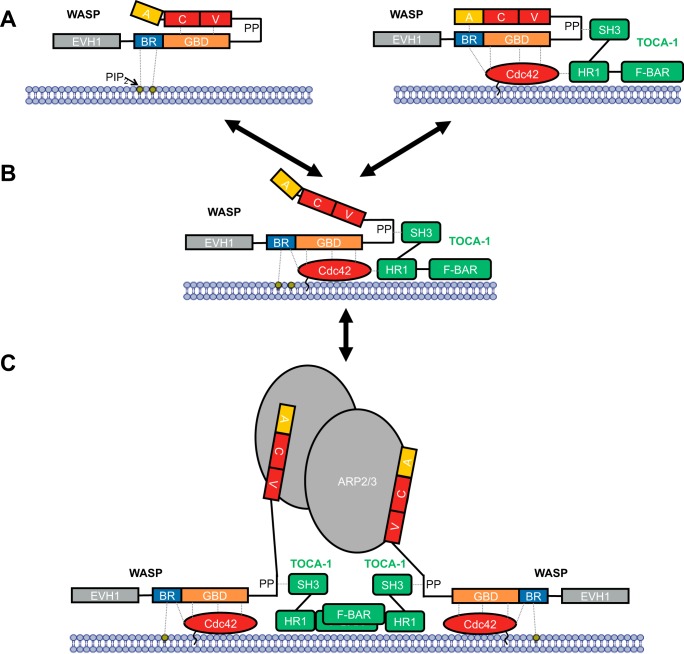
**Model for WASP activation.** WASP is represented as in Fig. 1, together with binding partners PIP_2_ (labeled, *yellow circle*), Cdc42 (*red oval*), TOCA1 (*green,* composed of domains SH3, HR1, and F-BAR), and Arp2/3 (*gray ovals*). The cell membrane is represented by a *blue* bilayer with which PIP_2_ and Cdc42 are associated. *A,* WASP binding to PIP_2_ (*left panel*) or WASP binding to Cdc42 (*right panel*) does not stimulate VCA release alone, but binding of both can relieve autoinhibitory interactions to release the VCA region. *B,* this can then bind to Arp2/3, which is fully activated by binding two VCA regions. *C,* dimerization through scaffolds such as TOCA1 can facilitate Arp2/3 activation by two VCA regions.

Arp2/3 binds two WASP VCA regions, and linked VCA dimers can hyperactivate the complex at concentrations below saturation, binding up to 180-fold more tightly (38). The combined actions of Cdc42 and PIP_2_ would lead to membrane localization of multiple WASP molecules, increasing the likelihood of two VCA regions being in sufficient proximity to engage Arp2/3 simultaneously. Clustering of the complexes into dimers/oligomers should more robustly induce Arp2/3 activity, and it is likely that this is tuned by either high PIP_2_ and Cdc42 densities or by a scaffold protein, for instance TOCA1 (transducer of Cdc42-dependent actin assembly), which exists in dimeric form and can engage two WASP proteins through the SH3–polyproline interaction, destabilizing the auto-inhibited structure (39, 40). TOCA1 can also bind to Cdc42; indeed a trimeric complex has been observed (41) despite the fact that TOCA1 and Cdc42 compete for WASP GBD binding (42). Nonetheless, TOCA1 links many of the common components required for robust Arp2/3 activation together. A model of filopodia formation is shown in Fig. 9, where the concerted action of TOCA1, PIP_2_, and active Cdc42 lead to activation of Arp2/3 by WASP. We envisage multiple input signals leading to local increased concentration of both PIP_2_ and activated Cdc42 at discrete membrane locations. Cdc42 would have the ability to bind TOCA1 via interaction with the HR1 domain of TOCA1, leading to incorporation of TOCA1 into the nanocluster. The concomitant actions of PIP_2_, Cdc42, and TOCA1 would lead to destabilization of the autoinhibited conformation of WASP. Local increased concentrations of active WASP would then drive dimeric recruitment of the Arp2/3 complex.

The work that we present here, and indeed many other published studies, makes use of nonphysiological systems, often *in vitro*, using truncated binding regions of component proteins. Binding constants and kinetic measurements are often made in the absence of membranes and membrane components, *e.g.* phospholipids. Nonetheless, putting all the data generated together gives a likely indication of the physiological processes involved in these complex systems.

### Role of the BR in other CRIB family effectors

PAK1, another CRIB effector of Cdc42, also exists in an autoinhibited conformation (involving GBD residues C-terminal to Asp-80, equivalent in position to WASP Gly-243) and possesses a BR (Fig. 8). Similarly, the CEP family of CRIB effectors possess BRs and are suspected to be subject to autoinhibition through the CII region (43). ACK, however, does not possess a BR and is not thought to be autoinhibited in a manner that involves the CRIB region (44). It appears that the presence of a BR in these effectors correlates with the formation of an autoinhibited structure involving the GBD, and the BR may therefore be a vital motif in breaking up the native tertiary structure in some CRIB effectors to ultimately allow GBD coalescence with Cdc42 and activation of the effector.

A new structure of full-length PAK4–Cdc42 also reports an interesting new potential function for the PAK4 BR (Fig. 8). In this structure, the PAK4 BR sits in the substrate-binding cleft of PAK4 in the presence of Cdc42, acting as an inhibitor in place of the displaced pseudosubstrate region (centered on Pro-52^WASP^) (15). These data may explain why Cdc42 does not significantly activate the kinase activity of the type II PAKs (45, 46); it seems rather that Cdc42 acts to prime PAK4 for full activation. If this complex were however situated at a membrane, phospholipid interactions could also be present to engage the PAK4 BR and fully activate the complex. Probably, the most informative structures will therefore stem from likely future analysis of CRIB effector complexes using full-length Cdc42 tethered to a membrane.

### Design of anti-Cdc42 peptidomimetics

The results we present here show the importance of the BR in defining the high affinity of WASP for Cdc42, because a truncated version of the WASP GBD with just 22 residues of the GBD remaining but with an intact basic region still binds with low nanomolar affinity and with a similar affinity to the entire ACK GBD. A peptidomimetic targeting this CRIB effector binding face of Cdc42 should thus benefit from the inclusion of a basic region, in particular a mimic of the C-terminal triad from the BR of WASP. As this is a positive charge density, it could also aid cell entry, providing a proverbial double-edged sword. The C-terminal GBD residues of WASP contribute less affinity than those in ACK and are thus dispensable in a potential therapeutic parent peptide. The ACK GBD is likely to prove an advantageous starting point, based on our studies on C-terminally truncated mutants of ACK and WASP; however, such a construct would greatly benefit from the inclusion of the C-terminal BR residues (equivalent to residues 230–232 of WASP).

## Conclusions

The central importance of specific WASP residues, particularly the consensus CRIB motif at the N-terminal GBD, in Cdc42 binding has been demonstrated in this work (Fig. 6). These data show that the contributions from many WASP residues are similar to those found for ACK in a previous study dissecting the ACK–Cdc42-binding interface. The major difference in binding between the two CRIB effectors lies in the BR, which, in WASP, is involved in electrostatic steering through interaction with an area of negative charge density on Cdc42. This brings the N terminus of the GBD close to the initiator subsite for coalescence at a more structurally fixed region of Cdc42 where docking can occur. This is followed by coalescence with the flexible switch regions of Cdc42, as in Cdc42–ACK binding. The residues of the BR can all contribute to electrostatic steering and increase the on-rate of binding; however, the C-terminal three residues are considerably more influential than the N-terminal trio. In addition, these N-terminal residues have no role in the bound complex, whereas the C-terminal residues, interestingly, also appear to contribute to the final complex with Cdc42 in a delocalized charge-dependent manner.

The soluble *in vitro* system studied here belies the complexity of WASP interactions in a physiological setting. Full-length WASP exists in an auto-inhibited conformation and has many activation partners of which PIP_2_ appears particularly synergistic with Cdc42. The BR has been observed to bind to PIP_2_, and it seems likely that this area of negative charge exchanges interactions with the acidic region of WASP and PIP_2_ (depending on local density). The BR is not neutralized by Cdc42 binding so it could still support an intramolecular interaction with the acidic region (Fig. 9). This together with the high on/off-rate of WASP binding explains the inability of Cdc42–WASP binding alone to destabilize WASP auto-inhibition sufficiently for Arp2/3 activation.

## Experimental procedures

### Protein expression constructs

All WASP–GBD peptides were expressed as GST fusion proteins in pGEX-2T (Amersham Biosciences) with residues 201–321 of WASP cloned into BamH1 and EcoR1 sites of the multicloning site (26). The construct expressing Cdc42 Δ7 Q61L (47) has been described previously.

### Mutagenesis

All targeted residues in WASP were mutated to alanine. Site-directed mutagenesis of WASP(201–321) was performed using the QuikChange Lightning multisite-directed mutagenesis kit (Agilent), and mutations were confirmed by sequencing (Department of Biochemistry, DNA Sequencing Facility, Cambridge, UK).

### Recombinant protein production

*Escherichia coli* BL21 cells were used to express all recombinant proteins. Stationary cultures were diluted in fresh 2TY growth medium (1:10) and grown to *A*_600_ 0.6–0.8 at 37 °C, whereupon they were induced with isopropyl β-d-1-thiogalactopyranoside (0.1 mm) for 5 h. Proteins were affinity-purified on GSH-agarose resin (Sigma). Cdc42 was cleaved from GST using thrombin (Novagen) and then further purified by gel filtration (S75 16/60, GE Healthcare). GST–WASP proteins for use in direct binding assays were eluted from the resin using 10 mm reduced GSH (in 50 mm Tris-HCl, 150 mm NaCl, pH 7.5). GST–WASP K230A, K231A, K232A, and K235A mutant proteins were further purified using size-exclusion chromatography (S200 16/60). Ion-exchange chromatography (Resource Q, GE Healthcare) was employed to purify the rest of the GST–WASP mutants. Protein concentrations were measured by *A*_280_ using calculated extinction coefficients.

### Nucleotide exchange

Cdc42 was labeled with [^3^H]GTP as follows. [^3^H]GTP (PerkinElmer Life Sciences) was dried by centrifugal evaporation, and 0.7 mg of Cdc42, 15 nm phosphoenolpyruvate, 6 units of pyruvate kinase (Sigma), 15 mm KCl, and 0.36 m (NH_4_)_2_SO_4_ were added in 140 μl of buffer (10 mm Tris-HCl, 150 mm NaCl, 1 mm DTT, pH 7.5). The mix was incubated at 37 °C for 3 h after which 10 mm MgCl_2_ was added to quench the reaction. Unbound nucleotide was removed with Sephadex G-25 spin columns (GE Healthcare).

### SPA

Affinities of WASP proteins for Cdc42 were measured using SPA. 5 nm individual WASP mutants were immobilized on protein A SPA fluoromicrospheres via an anti-GST antibody (Sigma). The equilibrium binding constants (*K_d_*) of the effector–G protein interaction were determined by monitoring the SPA signal in the presence of varying concentrations of [^3^H]GTP·Cdc42, as described previously (47). Binding of Cdc42 to the effector domain brings the radiolabeled nucleotide into proximity with the scintillant, allowing a signal to be generated. For each WASP mutant, a negative control was performed in the absence of effector, which resulted in a linear increase in background SPA counts. This dataset was then subtracted from the data points obtained in the presence of effector, and the adjusted values were plotted as a function of increasing concentrations of Cdc42. For each affinity determination, data points were obtained for at least 12 different G protein concentrations. Binding curves were fitted using a direct binding isotherm (48) using data points from all experimental replicates to obtain *K_d_* values and curve fitting errors for the G protein–effector interactions.

### Bio-layer interferometry

The interaction between Cdc42 and GST fusion constructs of WASP variants were studied using bio-layer interferometry on an Octet Red system (ForteBio). Experiments were performed in 50 mm Tris-HCl, pH 7.5, 150 mm NaCl, 1 mm MgCl_2_, 0.02% Tween 20, 0.1% BSA, and 0.05% sodium azide at 25 °C. Anti-GST sensors were loaded with GST fusion proteins at concentrations of 10 μg/ml, washed to return to baseline, and then dipped into a dilution range of Cdc42·GMPPNP to measure association. Dissociation was measured by dipping sensors into buffer after the association step. To minimize the effects of nonspecific binding, binding data were collected for sensors loaded with GST alone, and this was subtracted from the experimental traces. Sensors were regenerated in 10 mm glycine, pH 2, for reuse. Analysis was undertaken using the Octet Red analysis software.

## Author contributions

G. J. N. T., H. R. M., and D. O. formal analysis; G. J. N. T., H. R. M., and D. O. supervision; G. J. N. T., A. S., A. J. F., H. R. M., and D. O. investigation; G. J. N. T., A. S., A. J. F., H. R. M., and D. O. writing-original draft; G. J. N. T., H. R. M., and D. O. writing-review and editing; H. R. M. and D. O. conceptualization; D. O. funding acquisition; D. O. project administration.

## Footnotes

3 The abbreviations used are:

GEF: guanine nucleotide exchange factor
GAP: GTPase-activating protein
ACK: Cdc42-associated kinase
BLI: bio-layer interferometry
BR: basic region
CRIB: Cdc42/Rac interactive binding
GBD: G protein-binding domain
GST: glutathione *S*-transferase
PAK: p21-activated kinase
SPA: scintillation proximity assay
WASP: Wiskott–Aldrich syndrome protein
GMPPNP: guanosine-5′-[(β,γ)-imido]triphosphate
PDB: Protein Data Bank
PIP_2_: phosphatidylinositol 4,5-bisphosphate
SH: Src homology.
